# GLP-1 vasodilatation in humans with coronary artery disease is not adenosine mediated

**DOI:** 10.1186/s12872-021-02030-5

**Published:** 2021-05-01

**Authors:** Muhammad Aetesam-ur-Rahman, Joel P. Giblett, Bharat Khialani, Stephen Kyranis, Sophie J. Clarke, Tian X. Zhao, Denise M. Braganza, Sarah C. Clarke, Nick E. J. West, Martin R. Bennett, Stephen P. Hoole

**Affiliations:** 1 Department of Interventional Cardiology, Royal Papworth Hospital, Papworth Road, Cambridge Biomedical Campus, Cambridge, CB2 0AY UK; 2 Division of Cardiovascular Medicine, University of Cambridge, Cambridge, UK

**Keywords:** Glucagon-like peptide 1 (GLP-1), Glucagon-like peptide 1 receptor agonists (GLP-1 RA), Basal microvascular resistance (BMR), Index of microvascular resistance (IMR), Coronary artery disease (CAD)

## Abstract

**Background:**

Incretin therapies appear to provide cardioprotection and improve cardiovascular outcomes in patients with diabetes, but the mechanism of this effect remains elusive. We have previously shown that glucagon-like peptide (GLP)-1 is a coronary vasodilator and we sought to investigate if this is an adenosine-mediated effect.

**Methods:**

We recruited 41 patients having percutaneous coronary intervention (PCI) for stable angina and allocated them into four groups administering a specific study-related infusion following successful PCI: GLP-1 infusion (Group G) (n = 10); Placebo, normal saline infusion (Group P) (n = 11); GLP-1 + Theophylline infusion (Group GT) (n = 10); and Theophylline infusion (Group T) (n = 10). A pressure wire assessment of coronary distal pressure and flow velocity (thermodilution transit time—Tmn) at rest and hyperaemia was performed after PCI and repeated following the study infusion to derive basal and index of microvascular resistance (BMR and IMR).

**Results:**

There were no significant differences in the demographics of patients recruited to our study. Most of the patients were not diabetic. GLP-1 caused significant reduction of resting Tmn that was not attenuated by theophylline: mean delta Tmn (SD) group G − 0.23 s (0.27) versus group GT − 0.18 s (0.37), *p* = 0.65. Theophylline alone (group T) did not significantly alter resting flow velocity compared to group GT: delta Tmn in group T 0.04 s (0.15), *p* = 0.30. The resulting decrease in BMR observed in group G persisted in group GT: − 20.83 mmHg s (24.54 vs. − 21.20 mmHg s (30.41), *p* = 0.97. GLP-1 did not increase circulating adenosine levels in group GT more than group T: delta median adenosine − 2.0 ng/ml (− 117.1, 14.8) versus − 0.5 ng/ml (− 19.6, 9.4); *p* = 0.60.

**Conclusion:**

The vasodilatory effect of GLP-1 is not abolished by theophylline and GLP-1 does not increase adenosine levels, indicating an adenosine-independent mechanism of GLP-1 coronary vasodilatation.

*Trial registration:* The local research ethics committee approved the study (National Research Ethics Service-NRES Committee, East of England): REC reference 14/EE/0018. The study was performed according to institutional guidelines, was registered on http://www.clinicaltrials.gov (unique identifier: NCT03502083) and the study conformed to the principles outlined in the Declaration of Helsinki.

**Supplementary Information:**

The online version contains supplementary material available at 10.1186/s12872-021-02030-5.

## Background

Cardiovascular disease is the leading cause of death across the world, predominantly related to atherosclerosis [1]. Diabetes mellitus is one of the major risk factors for premature atherosclerotic coronary artery disease (CAD). Patients with diabetes mellitus are also susceptible to microvascular dysfunction. Endothelium-dependent vasodilation and microvascular coronary flow are frequently abnormal in patients with diabetes [2] and both are partly responsible for the observed increased cardiac morbidity and mortality in patients with diabetes mellitus.

Safety trials of contemporary hypoglycaemic treatments for type-2 diabetes mellitus have demonstrated beneficial reductions in cardiovascular outcomes [3–6]. One class of drugs—the incretin hormone, Glucagon-like peptide (GLP)-1, stimulates glucose-dependent insulin release and suppresses glucagon resulting in hypoglycaemia [7]. GLP-1, as well as its analogues, such as semaglutide and liraglutide, also improve long-term cardiovascular outcomes with reduction in myocardial infarction and cardiovascular death for patients with diabetes [3, 6, 8]. GLP-1 has been shown to improve left ventricular function during ischaemia–reperfusion injury [9, 10]. However, the underlying mechanism of these of target GLP-1 effects is not well understood [11], although the GLP-1 receptor is expressed in heart tissue and in particular on vascular smooth muscle cells [12].

Adenosine is a naturally occurring compound that binds to A2A and A2B receptors in the microcirculation [13], exerting a potent vasodilatory effect in vessels below 150 µm [14]. Our previous work has shown that GLP-1 causes coronary microvascular vasodilatation and increases coronary flow velocity in humans [15]. Animal studies have shown that alogliptin, an inhibitor of dipeptidyl peptidase (DPP)-4, a ubiquitous enzyme responsible for the degradation of active GLP-1(7–36) to GLP-1(9–36), exerts its cardioprotective effect on infarct size reduction via an adenosine receptor-dependent pathway [16]. A similar adenosine-dependent mechanism may be responsible for the cardioprotective effect of GLP-1 in humans, although this has not yet been explored.

We have undertaken a mechanistic study to explore whether GLP-1 causes coronary microvascular vasodilatation via an adenosine-mediated pathway in humans.

## Methods

### Identification and recruitment of patients (Fig. 1)

**Fig. 1:**
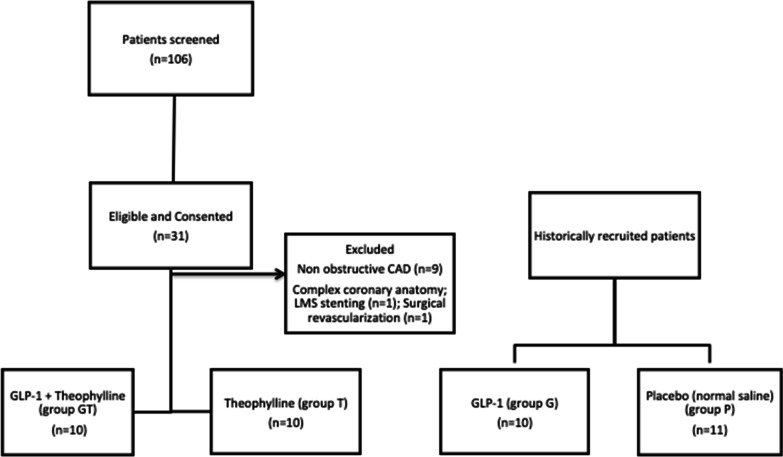
Consort diagram of the recruitment and allocation of study patients

One hundred and one patients with stable angina awaiting elective angiography were screened and thirty-one patients were found to be eligible and gave informed written consent. Out of these, nine patients had non-obstructive coronary arteries and therefore, did not require stenting. Two further patients had complex coronary anatomy: one requiring left main bifurcation stenting and the other had surgical revascularization, and therefore were excluded from our study.

Inclusion criteria included patients undergoing elective PCI for stable angina; age above 18 years; and able to give informed consent for the study. Exclusion criteria included any severe co-morbidity with expected life expectancy < 6 months; use of warfarin, nicorandil, glibenclamide, sitagliptin, vildagliptin, saxagliptin, linagliptin, alogliptin, exenatide, liraglutide, lixisenatide and insulin use; women of child-bearing age; breast-feeding women; myocardial infarction within the previous 3 months in a remote territory; heart failure with ejection fraction < 50%; deranged renal function with eGFR < 60 ml/min/1.73 m^2^ by Modification of Diet in Renal Disease (MDRD); deranged liver function with alanine transaminase (ALT) > 3 times upper limit of normal; active peptic ulcer disease confirmed on endoscopy; history of seizures; history of tachyarrhythmias; patients already taking oral theophylline; allergy to theophylline or caffeine.

Twenty patients having percutaneous coronary intervention (PCI) were studied in two groups: those receiving post-PCI infusions of GLP-1 + Theophyline (Group GT) and Theophylline (Group T) respectively. Data from these two groups were compared with historically-recruited patients who received GLP-1 infusion (Group G) and placebo (normal saline infusion) (Group P) [15]. Theophylline infusion was used with and without GLP-1 as an adenosine receptor antagonist to determine any adenosine mediated effect of GLP-1.

### Procedural details

All patients received aspirin, 300 mg and clopidogrel, 300 mg preloading, unless they were already established on these antiplatelets. Patients were anticoagulated with a heparin bolus (70–100 U/kg) after arterial sheath insertion (radial or femoral) to achieve an activated clotting time > 250 s. Iopromide (Ultravist; Bayer HealthCare Pharmaceuticals, Leverkusen, Germany) was used as the contrast agent for all cases. The choice of stent and implantation technique was left to operator discretion. Following successful stent implantation baseline bloods were taken to measure serum adenosine levels using a Stop solution.

A Pressure wire X (Abbott Vascular, Santa Clara), connected wirelessly to Coroflow (Coroventis, Uppsala), was positioned and maintained in the distal third of the stented coronary artery. A 0.2 mg bolus of intracoronary glyceryl trinitrate (GTN) was administered, and once steady state coronary haemodynamics were achieved, the baseline coronary pressures [aortic pressure (Pa) and distal wire pressures (Pd)] and flow velocity measurements were measured. The latter was derived from the reciprocal of mean transit time (Tmn) of an intracoronary injectate of room temperature saline (thermodilution technique) measured in triplicate [17, 18]. These measurements were repeated following intravenous administration of adenosine at 140 mcg/kg/min. Coronary wedge pressure (Pw) was measured separately as Pd during the occlusive coronary balloon inflation.

An intravenous infusion of GLP-1 (1.2 pmol/kg/min)(7–36) amide (Bachem AG, Switzerland) and an adenosine receptor inhibitor, theophylline (5 mg/kg in 100 ml 0.9% NaCl over 20 min) or GLP-1 or Theophylline (5 mg/kg in 100 ml 0.9% NaCl over 20 min) (Hameln pharma Ltd; UK) or Placebo (100 ml 0.9% NaCl over 20 min) was infused depending upon patient’s group. At the end of infusion, a repeat blood sample was taken from the coronary catheter to measure theophylline and adenosine levels. All the haemodynamic measurements were repeated at rest and hyperaemia after completion of the infusion, usually within 30-min of baseline. At the end of the procedure, the pressure wire was withdrawn to the coronary ostium to enable pressure-drift correction of Pd, if necessary. Pv was assumed to be 5 mmHg in all the patients in this study.

**Fig. 2:**
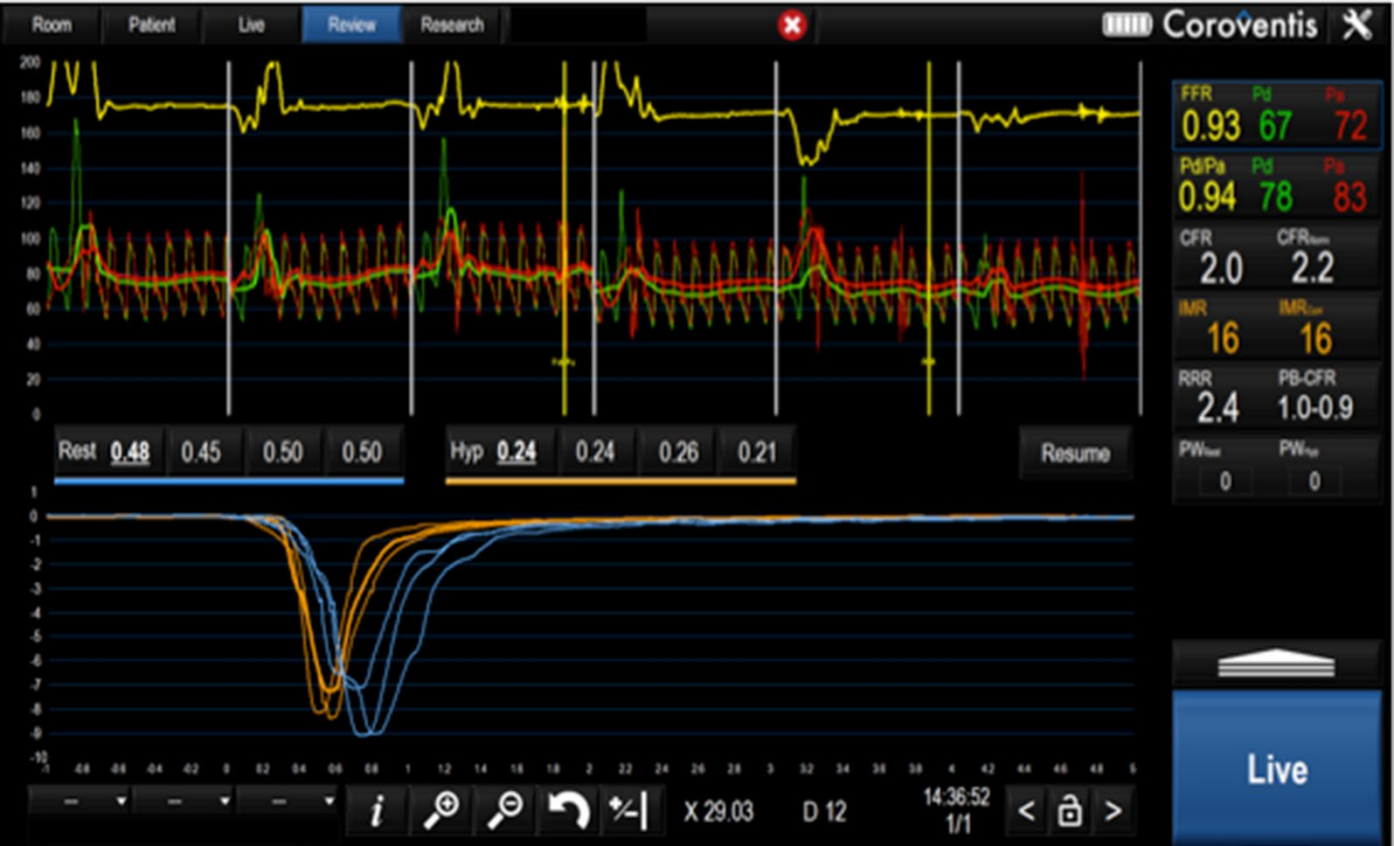
Example of Coroventis screen during invasive haemodynamic assessment of coronary artery. Blue curves show resting Tmn, orange showing hyperaemic Tmn. Resting as well as hyperaemic pressure and flow indices including Pa, Pd, FFR, IMR and CFR are displayed in the upper right-hand corner

These measurements enabled offline calculation of, basal microvascular resistance (BMR = Pa × Tmn × ((Pd − Pw)/(Pa − Pw))_baseline_) and index of microvascular resistance (IMR = Pa × Tmn × ((Pd − Pw)/(Pa − Pw)) _hyperaemia_), both corrected for collaterals, fractional flow reserve, (FFR = (Pd)/(Pa)_hyperaemia_), coronary flow reserve (CFR = (Tmn) _baseline_/(Tmn)_hyperaemia_) and collateral flow index by pressure (CFI_P_ = (Pw − Pv)/(Pa − Pv)_baseline_) and coronary resistive reserve ratio (RRR = BMR/IMR), as previously described and validated [19, 20] (Fig. 2).

### Blood sampling

Adenosine has a very short half-life and therefore we used a previously published composition of Stop solution to prevent enzymatic breakdown of the extracted serum adenosine samples [21, 22] Blood samples to determine adenosine concentration were collected from the guide catheter, at the completion of PCI and after 20 min of study drug infusion, and placed directly into vacutainers containing Stop solution. This comprised dipyridamole 0.2 mmol/L, 4.2 mmol/L ethylene-diamine-tetraacetic acid disodium (Na2 EDTA), erythro-9-(2-hydroxy-3-nonyl)-adenine (EHNA) 5 mmol/L, α,β-methyleneadenosine-5′-diphosphate (AMPCP) 79 mmol/L, heparin sulfate 1 IU/mL, deoxycoformycin 1 μg/mL in 0.9% NaCl, all sourced from Merck, UK. After centrifugation, supernatants were deproteinized, and serum adenosine concentration measured by high-performance liquid chromatography. Similarly, blood for theophylline levels was collected from coronary arteries just before the end of infusion and analyzed to confirm therapeutic levels.

### Statistical analysis

On the basis of previous data, we calculated that 10-paired data sets would provide 80% power to detect a clinically significant difference (ΔBMR, 20 mmHg s; SD, 15 mmHg s) after administration of GLP‐1.

Data are given as mean (SD) or median (Q1, Q3) as appropriate unless otherwise stated. Comparisons were made for any significant differences by unpaired T test, one-way ANOVA or Kruskal–Wallis test, where appropriate using GraphPad Prism version 8.1.2 (227) (GraphPad Software, La Jolla California USA). Similarly, a simple linear regression was performed between resting adenosine levels and basal coronary flow velocity before and after the study infusion to explore any correlation. A two-sided value of *p* < 0.05 was deemed significant. Authors had full access to the data and take full responsibility its integrity.

## Results

### Baseline characteristics

Baseline characteristics are summarized in Table 1. All four groups (G, P, GT and T) were well matched although the GT group had more female patients and fewer patients receiving GLP-1 were taking an ACE-inhibitor or angiotensin receptor blocker. Of note, the majority of patients recruited into this study were not diabetic.

**Table 1:** Baseline characteristics

	GLP-1 group	Saline group	GLP-1 + Theophylline group	Theophylline group	*p* value
Age	68.30 (10.13)	62.30 (7.44)	62.20 (8.19)	63.10 (18.39)	0.62
Male	8 (80)	11 (100)	4 (40)	8 (80)	**0.002**
Diabetes	1 (10)	2 (18)	0(0)	1 (10)	0.57
Hypertension	6 (60)	8 (73)	4 (40)	5 (50)	0.31
Previous MI	1 (10)	8 (73)	4 (40)	3 (30)	0.06
(Ex) Smoker	7 (70)	7 (64)	2 (20)	7 (70)	0.09
Statin	10 (100)	11 (100)	10(10)	10 (10)	1
ACEI/ ARB	3 (30)	7 (64)	4 (40)	8 (80)	**0.03**
B Blocker	4 (40)	8 (73)	7 (70)	9 (90)	0.13

Data is given as Mean (SD) and n (%) where appropriate. *p* < 0.05 is considered significant. *p* < *0.05* is given as bold

### Haemodynamic data

**Fig. 3:**
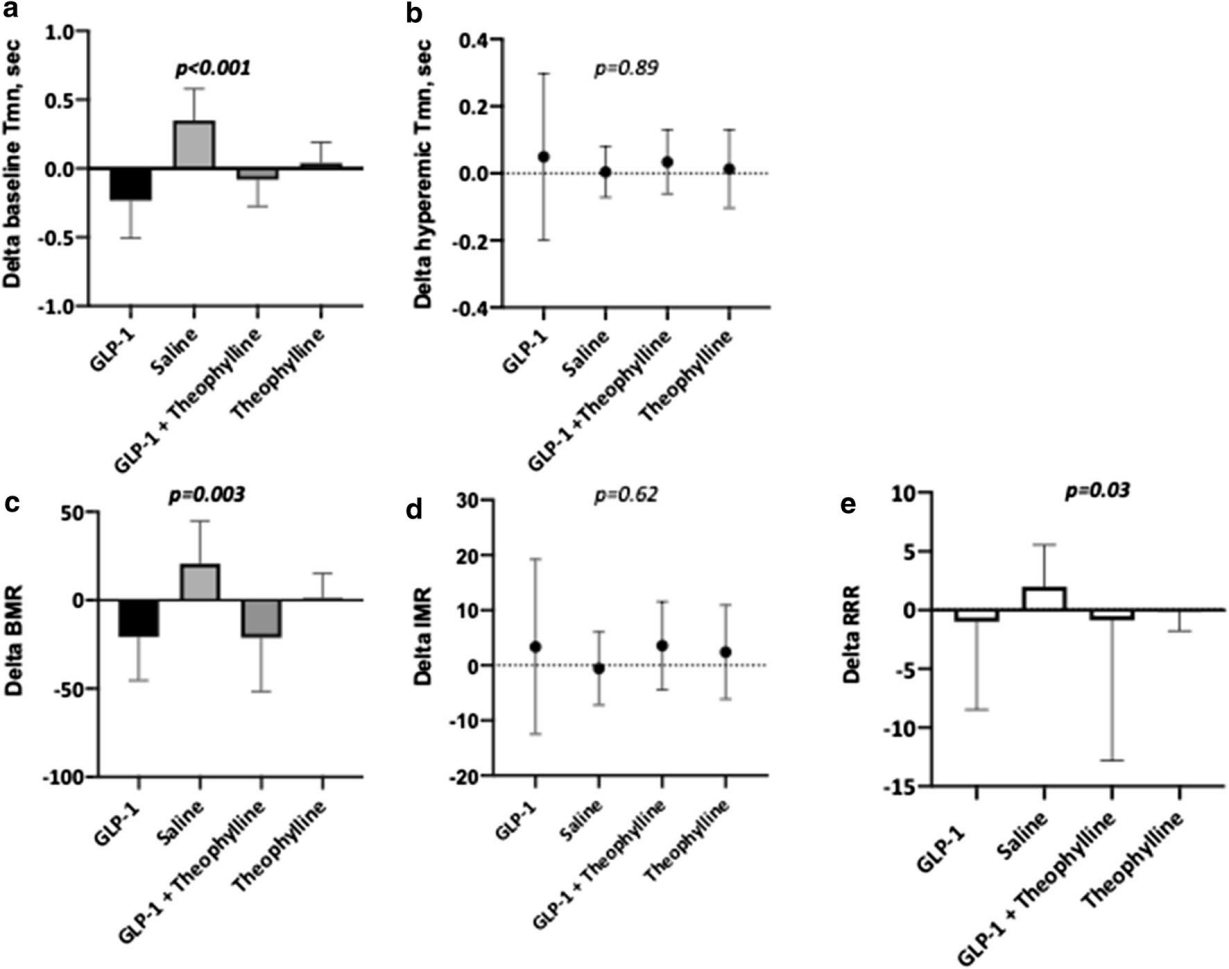
Comparison of change in thermodilution time, Tmn at rest (**a**) and hyperemia (**b**); basal microvascular resistance, BMR (**c**); index of microvascular resistance, IMR (**d**) and resistive reserve ratio, RRR (**e**) after each infusion*. p* < ***0.05 ***is given as bold

Haemodynamic data are summarized in Table 2 and illustrated in Fig. 2. There were no differences in the baseline or post-infusion heart rate and blood pressure between all the four groups. Similarly, there were no differences in CFR and FFR immediately after stenting and following infusion. The basal microvascular resistance (BMR) before infusion was similar in all groups. BMR was significantly lower in the groups receiving GLP-1 (G and GT) after infusion compared to baseline: delta BMR − 20.83 (24.8) and − 21.20 (30.1), *p* = 0.97 respectively, confirming that theophylline did not attenuate the GLP-1 vasodilatory effect in group GT (Fig. 2). The lower BMR was attributed to a significantly lower (faster) resting Tmn after infusion in the G and GT groups: − 0.23 (0.27) and − 0.18 (0.37) respectively, whereas it was essentially unaltered in group T, 0.04 (0.15), *p* < 0.001. GLP-1 exhausted the vasodilatory capacity of the microvasculature, with delta median resistive reserve ratio (RRR) in group G: − 1.00 (− 2.95, − 0.01) and in group GT: − 0.88 (− 3.79, − 0.27) compared to 2.01 (0.23, 3.57) in group S and − 0.16 (− 1.06, 0.61) in group T, *p* = 0.03, (Fig. 3). Similarly, delta CFR was lower in group G: − 0.89 (− 2.01, 0.97) versus the other three groups. There was no significant difference in IMR or delta IMR across all the four groups.

**Table 2:** Haemodynamic data of study patients

Variable	GLP-1 group	Saline group	GLP-1 + Theophylline group	Theophylline group	*p* value
Immediate post PCI					
*Baseline*					
Heart rate (bpm)	65.6 (8.9)	79.3 (32.1)	68.5 (9.0)	62.6 (4.6)	0.21
Systolic BP (mmHg)	131.8 (27.5)	133.1 (35.9)	138.3 (20.3)	133.4 (25.7)	0.91
Diastolic BP (mmHg)	59.2 (11.3)	72.3 (17.1)	64.8 (9.4)	66.3 (9.9)	0.14
Pa mmHg	89.7 (16.5)	97.3 (23.4)	95.1 (10.8)	97.6 (14.9)	0.58
Pd mmHg	84.6 (16.5)	93.3 (24.1)	90.5 (9.6)	94.3 (15.1)	0.49
Pd/Pa	0.94 (0.04)	0.95 (0.04)	0.95 (0.04)	0.96 (0.04)	0.51
Tmn (s)	0.87 (0.39)	0.48 (0.23)	0.85 (0.79)	0.48 (0.40)	0.16
BMR	76.3 (37.9)	45.9 (34.7)	78.5 (70.8)	46.6 (44.8)	0.25
*Hyperaemia*					
Pa (mmHg)	81.2 (17.8)	90.6 (19.9)	89.9 (14.1)	86.2 (17.3)	0.49
Pd (mmHg)	71.9 (14.9)	81.6 (19.7)	80.0 (11.7)	79.5 (16.0)	0.44
FFR	0.88 (0.06)	0.89 (0.08)	0.89 (0.05)	0.92 (0.07)	0.41
Tmn (s)	0.24 (0.10)	0.20 (0.07)	0.21 (0.10)	0.24 (0.17)	0.71
CFR	4.0 (2.2)	2.4 (0.8)	4.6 (4.2)	2.1 (0.8)	0.16
IMR	16.3 (10.2)	15.6 (5.8)	16.5 (9.7)	18.8 (14.2)	0.86
CFIP	0.15 (0, 0.23)	0.09 (0, 0.21)	0.12 (0.05, 0.27)	0.18 (0.06, 0.29)	0.78
RRR	5.1 (1.9)	2.9 (1.3)	5.5 (5.4)	2.6 (1.2)	0.13
Post Infusion					
*Baseline*					
Heart rate (bpm)	63.0 (13.9)	68.7 (11.9)	68.8 (8.4)	66.0 (12.3)	0.58
Systolic BP (mmHg)	138.3 (22.3)	140.1 (26.1)	134.7 (21.5)	133.0 (23.1)	0.81
Diastolic BP (mmHg)	63.4 (7.8)	71.8 (15.3)	68.3 (13.2)	64.8 (13.1)	0.36
Pa (mmHg)	90.8 (16.6)	89.6 (15.4)	92.6 (15.3)	94.9 (8.6)	0.77
Pd (mmHg)	84.9 (15.9)	86.4 (16.7)	88.6 (14.2)	91.4 (9.0)	0.65
Pd/Pa	0.93 (0.03)	0.96 (0.04)	0.96 (0.04)	0.96 (0.03)	0.11
Tmn (s)	0.63 (0.27)	0.83 (0.41)	0.66 (0.54)	0.52 (0.39)	0.37
BMR	55.4 (30.4)	66.7 (37.2)	57.3 (48.5)	47.8 (38.8)	0.64
*Hyperaemia*					
Pa (mmHg)	80.1 (16.7)	89.6 (15.4)	90.5 (10.9)	89.5 (23.9)	0.38
Pd (mmHg)	71.9 (15.3)	76.2 (17.1)	88.6 (14.2)	82.6 (19.2)	0.14
FFR	0.89 (0.06)	0.89 (0.08)	0.93 (0.03)	0.93 (0.06)	0.24
Tmn (s)	0.29 (0.21)	0.21 (0.07)	0.25 (0.14)	0.26 (0.13)	0.53
CFR	3.0 (2.4)	4.2 (2.0)	2.6 (1.5)	2.1 (1.1)	0.11
IMR	19.7 (14.6)	15.0 (6.2)	20.1 (11.4)	21.2 (12.2)	0.49
CFIP	0.17 (0.00, 0.29)	0.08 (0.00, 0.23)	0.13 (0.05, 0.29)	0.19 (0.07, 0.28)	0.96
RRR	3.61 (2.5)	4.73 (2.3)	2.76 (1.6)	2.46 (1.5)	0.09

Results expressed as mean (SD). Tmn—transit time; Pa—aortic pressure; Pd– distal coronary pressure; BMR—basal microcirculatory resistance = Pa * Tmn _baseline_ * ((Pd − Pw)/(Pa − Pw)); IMR—index of microcirculatory resistance = Pa * Tmn_hyperemic_ * ((Pd − Pw)/(Pa − Pw)); FFR– fractional flow reserve = Pd/Pa_hyperemic_; CFR—coronary flow reserve = Tmn_baseline_/Tmn_hyperemic_; CFI_P_—Collateral flow index by pressure = (Pw − Pv)/(Pa − Pv)_baseline_ and coronary resistive reserve ratio (RRR) = BMR/IMR

### Biochemical data

The mean theophylline levels measured at the end of infusion were in the therapeutic range (10–20 mcg/ml) and similar between GT and T groups: 13.58 mcg/ml (4.48) versus 15.05 mcg/ml (2.15), *p* = 0.40. There was no difference in delta median adenosine levels after infusion between the two groups: GT, − 2.00 ng/ml (− 117.1, 14.8) versus T, − 0.50 ng/ml (− 19.6, 9.4), *p* = 0.60.

### Correlation of basal adenosine and Tmn

There was no correlation found in the measured adenosine levels and basal coronary flow velocity, before or after the study infusion (Additional file 1).

## Discussion

This study demonstrates that GLP-1 does not increase circulating adenosine levels, and that GLP-1-induced reduction in Tmn and BMR at rest was not attenuated by co-administration of the adenosine receptor antagonist theophylline. This indicates that GLP-1 exerts an adenosine-independent vasodilatatory effect.

GLP-1 receptor agonists are associated with improved long-term cardiovascular outcomes [6, 23] and a variable reduction in infarct size in previous human studies [8, 9, 24, 25]. Adenosine is a naturally occurring vasodilator and is a cellular mediator of cardioprotective ischaemic conditioning (IC) [26] by directly activating phospholipase C and/or protein kinase C (PKC) via adenosine 1 receptors (A1R), which are widely present in myocardial tissue [27, 28]. GLP-1 activates protein kinase A (PKA) along with other physiologically active metabolites at a cellular level [29]. The cross talk between PKC and PKA is well established and activation of PKA could in theory result in reduction of the activation threshold of PKC, thus potentiating the cardiac effects of adenosine [30, 31]. This PKC and PKA interaction has been postulated to be the underlying physiological mechanism of adenosine-mediated cardioprotection by GLP-1 in an animal model [16].

We have previously shown that GLP-1 attenuates ischaemia-induced LV dysfunction and the derived cardioprotection, but unlike conditioning is not associated with a potassium adenosine tri-phasphate (KATP) channel-dependent pathway [10] and is also independent of changes in cardiac substrate use [9]. More recently, we have shown that GLP-1 is a coronary vasodilator, possibly resulting indirectly from lusitropic forces “opening” the myocardial microcirculation in diastole as a result of ventricular–microcirculatory interactions [15, 32, 33]. Although in the same study we confirmed GLP-1 receptor(R) expression on ventricular myocytes, others have suggested that GLP-1R expression is confined to atrial cardiomyocytes [34] and vascular smooth muscle [12]. Therefore, we were keen to explore if GLP-1 could cause vasodilatation via an adenosine-mediated effect on the microcirculation, indirectly improving ventricular function via the Gregg effect [32].

We clearly demonstrate in this study that GLP-1 vasodilatation is unlikely to be the mediated by adenosine. GLP-1 increases basal coronary flow velocity and reduces BMR irrespective of theophylline. GLP-1 exhausts vasodilatory capacity, such that response to exogenous adenosine is blunted as measured by a reduction in CFR and RRR. Theophylline has been used to investigate adenosine-mediated effect of other therapies; it is a potent adenosine receptor inhibitor at levels achieved in our study [35]. In addition, the adenosine levels remained unchanged after GLP-1 infusion.

Coronary microvascular dysfunction (CMD) is associated with worse clinical outcomes [36] and microvascular injury at the time of elective PCI is associated with procedure-related myocardial infarction and a worse prognosis [37]. GLP-1 improves coronary flow after stenting [15], decreases periprocedural left ventricular dysfunction and stunning [8, 38, 39], and improves immediate as well as long-term cardiovascular outcomes after revascularization for coronary ischemia [24]. GLP-1 and its analogues should be further investigated for symptomatic as well as prognostic benefit in patients with CMD. Furthermore; GLP-1 has the potential to be a much simpler addition to the currently utilized armamentarium of cardioprotective strategies for patients at high risk of peri-procedural cardiovascular events [11].

## Limitations

There are several limitations in our study. Firstly, this was not a randomized controlled trial but was performed in two phases by block allocation to understand the mechanistic effects of GLP-1 on coronary physiology. However, the patients were unselected and sequentially recruited if eligible, and we believe this prevented significant bias. Second, we studied the coronary vasodilatory effects of GLP-1 following PCI. Coronary physiology may not be stable at this time due to reactive hyperaemia and microvascular stunning [15, 40]; however, we mitigated this by waiting for reactive hyperaemia to dissipate before making our measurements. Third, we used a surrogate for coronary flow—Tmn measured by a pressure wire based coronary thermodilution technique. This is a well-validated and accurate technique, that is comparable to other measures of coronary flow velocity [17, 18]. Fourth, we did not perform invasive measurements to confirm our previously published protective effects of GLP-1 on peri-procedural LV dysfunction and also assumed Pv to be 5 mmHg in our patients; this was for logistical reasons. Fifth, patients with diabetes were under-represented in our study and the GLP-1 effect in this group needs confirming. Finally, we only measured adenosine levels in the latter two prospectively-recruited GT and T groups due to logistical reasons. Endogenous adenosine levels did not correlate with resting coronary flow velocity. The reason for this is unclear but may be due to difficulties in assaying adenosine and also that the sample site was at the level of the coronary ostium rather than at the microcirulation. Theophylline is reported to increase local serum catecholamine levels by off-target effects, which may also blunt adenosine mediated vasodilatation [41]. It is also possible that different batches of Stop solution may explain some of the intergroup differences in adenosine levels, although patient-level changes in adenosine level were assayed with the same Stop solution and remain valid.

## Conclusion

The coronary vasodilatory effect of GLP-1 appears to be independent of adenosine. Further studies are required to understand the mechanism of the cardioprotective effects of GLP-1.

## Supplementary Information

**Additional file 1: Supplemetary Figure**. Sactterplot of the relationship between A) pre and B) post infusion adenosine concentration and Tmn_rest_.

## Data Availability

The datasets used and/or analysed during the current study are available from the corresponding author on reasonable request.

## Abbreviations

GLP-1: Glucagon-like peptide-1
GLP-1 RA: Glucagon-like peptide 1 receptor agonists
DPP-4: Dipeptidyl peptidase-4
PCI: Percutaneous coronary intervention
CAD: Coronary artery disease
CMD: Coronary microvascular dysfunction
MDRD: Modification of Diet in Renal Disease
ALT: Alanine transaminase
GTN: Glyceryl trinitrate
Tmn: Thermodilution transit time
Pa: Aortic pressure
Pd: Coronary distal pressures
Pw: Coronary wedge pressure
Pv: Central venous pressure
CFR: Coronary flow reserve
RRR: Resistive reserve ratio
BMR: Basal microvascular resistance
IMR: Index of microvascular resistance
