# Supplementary figures and images for: GLP-1 vasodilatation in humans with coronary artery disease is not adenosine mediated
Source: BMC Cardiovasc Disord. 2021 May 1;21:223. doi: 10.1186/s12872-021-02030-5 (PMC8088691; doi:10.1186/s12872-021-02030-5)

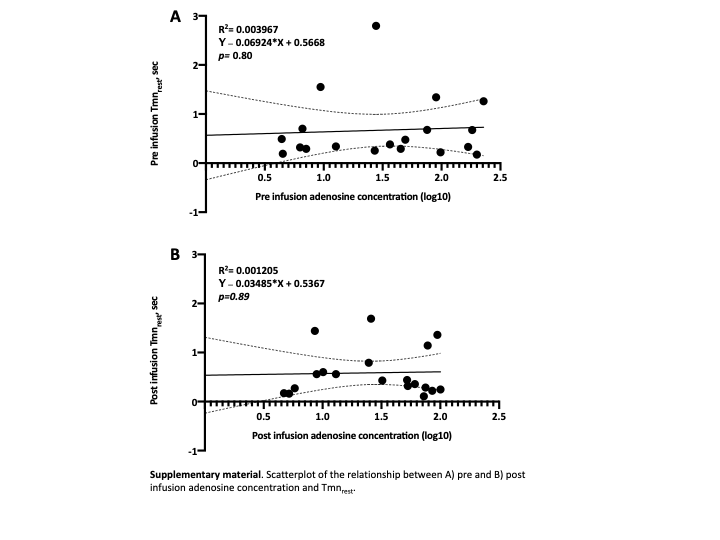

Supplement: Supplementary file 1 — Additional file 1: Supplemetary Figure. Sactterplot of the relationship between A) pre and B) post infusion adenosine concentration and Tmnrest. [file 12872_2021_2030_MOESM1_ESM.tiff]
